# Radio-pathological diagnosis of a retroperitoneal cavernous hemangioma

**DOI:** 10.1093/jscr/rjad095

**Published:** 2023-03-07

**Authors:** Ahmed S Khazaal, Omar M Sultan, Inas A A M Rasheed

**Affiliations:** Department of Surgery, College of Medicine, Tikrit University, Tikrit, Iraq; Department of Radiology, College of Medicine, Tikrit University, Tikrit, Iraq; Department of Pathology, College of Medicine, Tikrit University, Tikrit, Iraq

**Keywords:** Hemangioma, Retroperitoneal Space, Abdominal mass

## Abstract

Retroperitoneal cavernous hemangioma (RCH) is a rare benign vascular malformation. Only a few cases of RCH were reported. Here we present a case of RCH in a 66-year-old female complaining of long-standing progressive dull abdominal pain.

## INTRODUCTION

A 66-year-old female was visiting our teaching hospital complaining of long-standing dull abdominal pain radiating to the back, which become progressive and more evident in the last few months.

She had a chronic history of hypertension and was on regular treatment. She underwent left hip replacement surgery for years, otherwise is no recent history of surgical intervention or recent trauma.

A palpable mass was felt by clinical examination, and then the patient was sent for a *trans*-abdominal ultrasound examination, which confirms the presence of a left para-aortic large retroperitoneal mass lesion; then we requested a contrast-enhanced CT scan of the abdomen and pelvis.

## CASE REPORT

**Figure 1:**
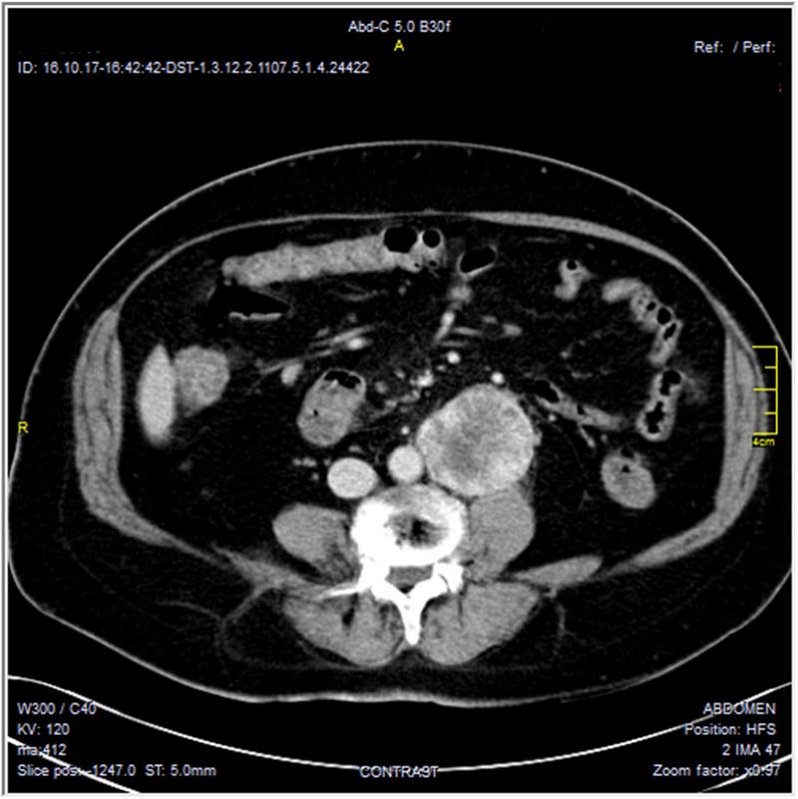
Contrast-enhanced axial CT images—soft tissue window, show well-defined peripherally enhancing mass lesion at left para-aortic region. No local invasion.

**Figure 2:**
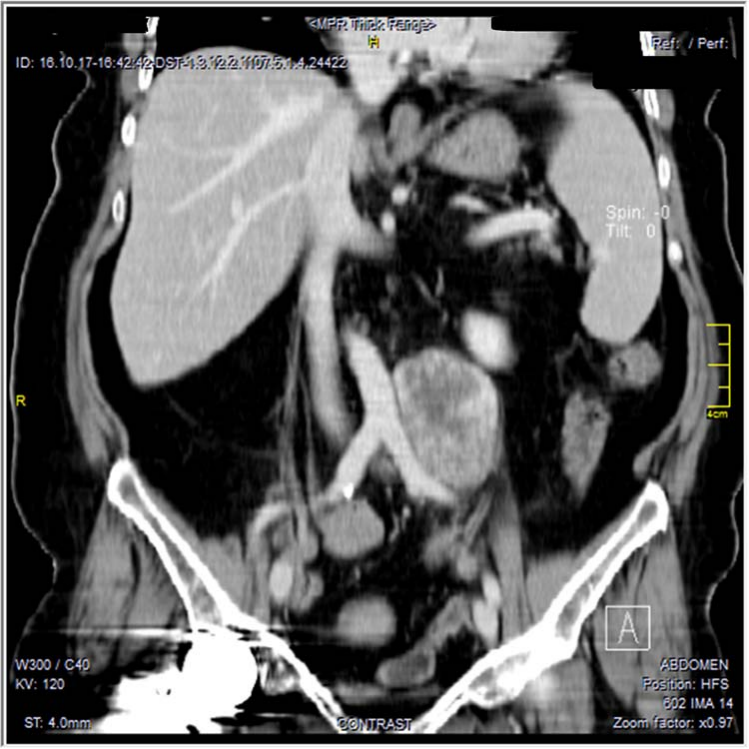
Contrast-enhanced reformatted coronal images—soft tissue window, show well-defined peripherally enhancing mass lesion seen along the left lateral wall of distal aorta and left common iliac artery. Preserved fat planes between the lesion and adjacent aorta and common iliac can be well appreciated with no signs of local invasion. A metal artifact from right hip prostheses is noted.

**Figure 3:**
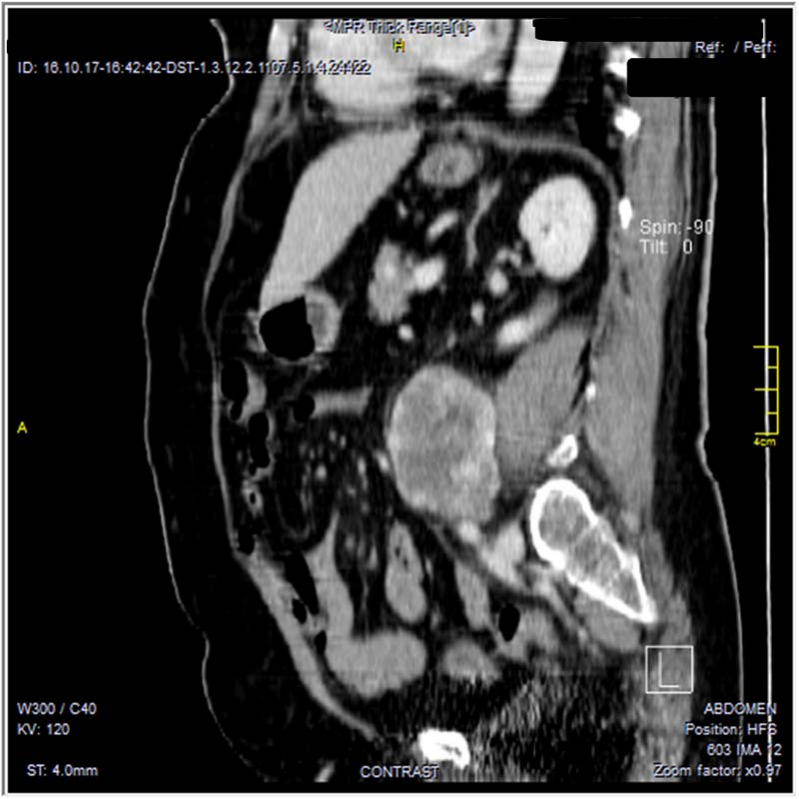
Contrast-enhanced reformatted sagittal images—soft tissue window, show well-defined peripherally enhancing lobular mass lesion. Preserved fat planes around the lesion are noted with no signs of local invasion.

The CT scan was confirming the presence of a relatively well-defined retroperitoneal lobulated mass lesion seen extending along the left lateral wall of the distal abdominal aorta and left common iliac artery and anteromedial to the left psoas muscle. It showed heterogeneous intense peripheral enhancement during the porto-venous phase, the fat planes were preserved between the lesion and adjacent structures with no gross evidence of local invasion (Figs 1–3). The first diagnostic possibility was made as a retroperitoneal paraganglioma based on the CT appearance. The surgical decision of tumor removal was done.

**Figure 4:**
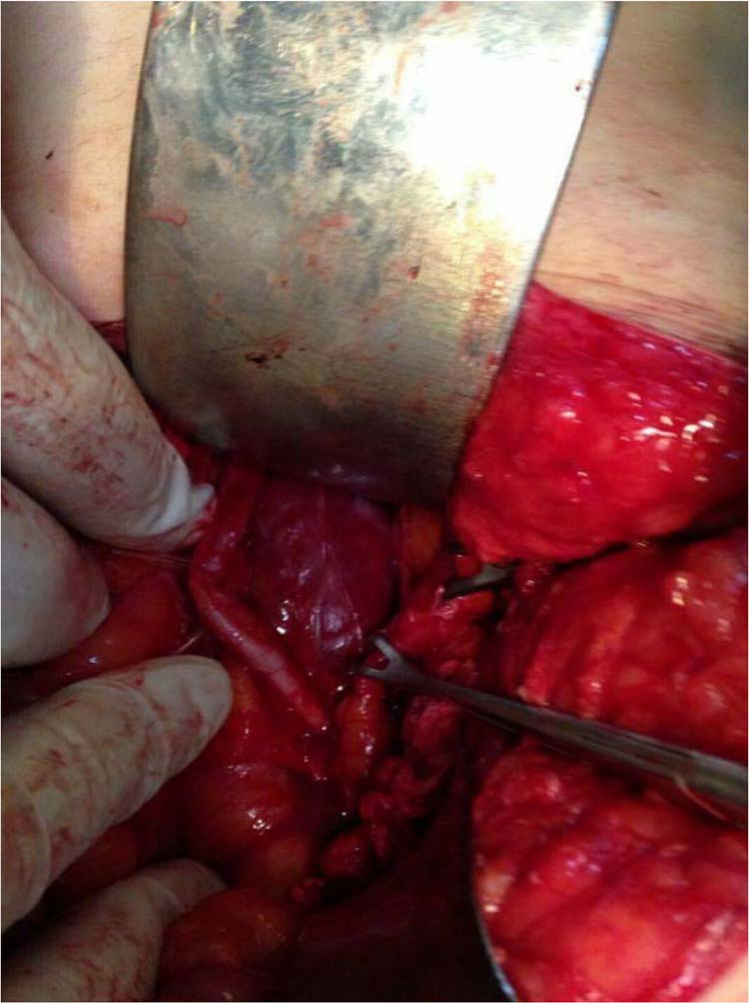
Intraoperative photo, showing the mass and ureter.

**Figure 5:**
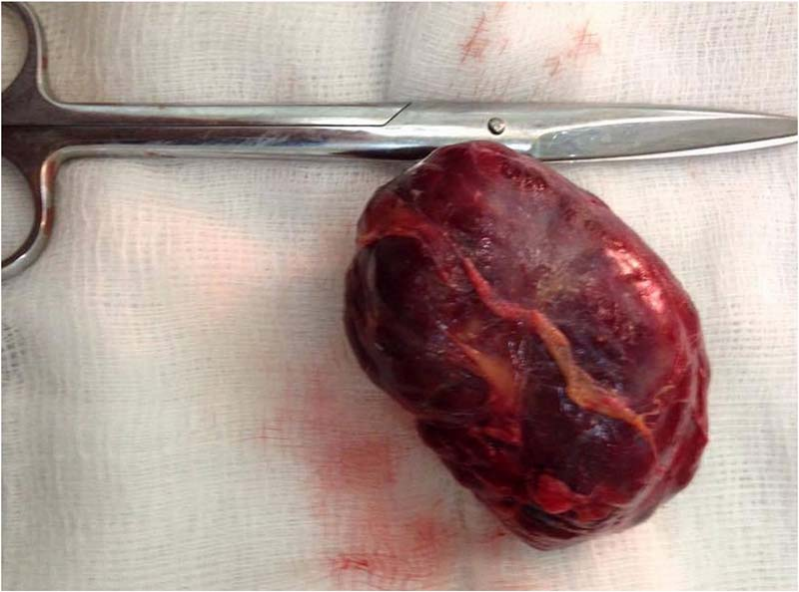
Postoperative photo shows well-defined lobulated dark mass.

Surgical resection of the tumor was performed using a lower midline incision and a Kocher maneuver was used for the dissection of the retroperitoneum. The mass was found, lying posterior to the left ureter and gonadal vessels (both of them isolated by a rubber tube) and compressing medially on the aorta above the bifurcation. The feeding arteries of the tumor were found to originate from retroperitoneal tissue instead of from the abdominal aorta, and each vessel was ligated before the tumor was removed. There was no evidence intraoperatively of the invasion of the surrounding structures. The mass was well capsulated showing a lobulated surface and dark fleshy color (Figs 4 and 5). The postoperative period was uneventful, and the patient was discharged home on Day 3 after the operation.

**Figure 6:**
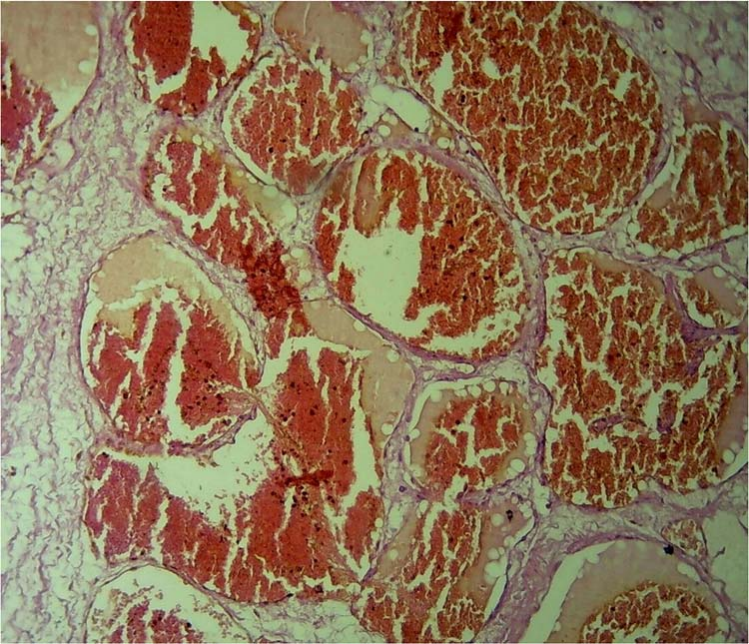
H&E-stained histological sections show hugely ectatic variable sizes vascular spaces lined by flattened endothelial cells filled with blood, separated by fibrous tissue and extending between the fatty tissues. Magnification power: ×40. Diagnosis: cavernous hemangioma.

The histopathology shows a well-capsulated mass composed of multiple variable sizes dilated vascular spaces consistent with a benign vascular tumor (cavernous hemangioma) with no evidence of atypia or malignancy (Fig. 6).

## DISCUSSION

Hemangioma is a benign vascular tumor that may involve the skin, mucosa, or viscera such as the liver and spleen. It rarely involves the retroperitoneal organs such as kidneys, pancreas, and adrenal glands [1–4].

Retroperitoneal hemangiomas are of the cavernous type and are commonly seen in pediatrics [5].

Adult hemangiomas are distinct from pediatric hemangiomas, which proliferate during infancy and then involute slowly for several years, followed by eventual regression [2].

Primary retroperitoneal cavernous hemangioma (RCH) is an extremely rare type of hemangioma [2], also it is considered a rarer tumor among the retroperitoneal neoplasms [1, 2]. Since 1950, only a few cases reported in the literature [1].

RCH are usually asymptomatic [1]. The main presenting symptom is dull abdominal pain, which would be because of the slow progressive growth of RCH. However, large RCHs can compress the surrounding organs and structures and may cause complications, such as hydronephrosis [2].

RCH can cause local invasion of adjacent structures and lead to aggressive symptoms and complications [3]. Increased growth of hemangiomas had led to internal thrombosis and obstruction from inadequate venous drainage [1, 6]. However, RCH may rupture, resulting in life-threatening hemorrhage requiring urgent surgical intervention [1, 7].

Our patient was an old-aged female. In general, no sex predilection had been found for RCH, except for adrenal glands, which are more common in women with a female:male ratio of ~2:1 [8–10].

Physical abdominal examination may reveal a palpable mass when it becomes large enough as in our case. Laboratory studies usually provide no additional indications for RCH [2].

It is difficult to make a diagnosis of RCH even by various imaging modalities and the diagnosis of all reported cases is made by histopathological examination [1].

However, the imaging findings of RCH may differ depending on the organ of origin, but in general, it shows well-defined hypoechoic and homogeneous lobulated lesions with positive internal vascularity by ultrasound examination [12]. On CT and MRI, it appears well defined with round or lobular margins, and with low attenuation (by CT), low signal intensity on T1-weighted imaging and high signal intensity on both T2-weighted imaging and heavily T2-weighted imaging relative to skeletal muscle. Calcified phleboliths when visualized have very low signal intensity on both T1-weighted imaging and T2-weighted imaging, are better visualized on CT and are strongly suggestive of the diagnosis. Non-parenchymal hemangiomas in the abdomen usually demonstrate progressive enhancement [11, 12]. However, MRI is the definitive diagnostic modality [5].

In our case, the US and CT imaging characteristics were benign and near classical to RCH, so the differential diagnosis of benign retroperitoneal mass lesion was made including paraganglioma, neurofibroma, lipoma, teratoma and neurilemoma [13]. Paraganglioma is considered the most possible diagnosis based on imaging findings. Other differential diagnoses of a malignant lesion such as liposarcoma, malignant fibrous histiocytoma, neuroblastoma and leiomyosarcoma may be considered but it is less likely [1, 2].

Operative and nonoperative approaches are possible for RCH patients. Because of the complexity of retroperitoneal anatomy, RCHs often involve vital structures, making resection extremely technically challenging, and high-volume centers are required for optimal opportunities for surveillance and multidisciplinary care [1, 9].

In our case, the tumor was localized and there was no evidence of local invasion or metastasis, we performed curative resection of the tumor to relieve the patient’s symptoms and to confirm the diagnosis of the benign lesion by histopathology.

## CONCLUSION

RCH is a rare adult mass lesion. The clinical diagnosis is difficult and imaging findings may also not be conclusive.

The optimal management of RCH has not yet been standardized and based on the lesion location, size, growth rate, local effect and patient’s symptoms. However, surgical resection is a curative choice for RCH. It reduces the risk of hemorrhage and relieves the pressure effect and patient’s symptoms.

## CONFLICT OF INTEREST STATEMENT

None declared.

## FUNDING

None.
